# Tracing the origin of Argentine Malbec wines by sensometrics

**DOI:** 10.1038/s41538-024-00252-3

**Published:** 2024-02-21

**Authors:** Roy Urvieta, Hildegarde Heymann, Annegret Cantu, Aníbal Catania, Fernando Buscema, Rubén Bottini, Ariel Fontana

**Affiliations:** 1 Grupo de Bioquímica Vegetal, Instituto de Biología Agrícola de Mendoza CONICET-UNCuyo, Almirante Brown 500, Chacras de Coria, Mendoza, M5507 Argentina; 2 Catena Institute of Wine, Bodega Catena Zapata, Cobos s/n, Agrelo, M5509 Argentina; 3 Department of Viticulture and Enology, University of California Davis, 595 Hilgard Lane, Davis, CA 95616 USA; 4 Estación Experimental Agropecuaria Mendoza, Instituto Nacional de Tecnología Agropecuaria, San Martín 3853, M5507 Av. Rivadavia, Argentina; 5 Instituto de Veterinaria, Ambiente y Salud, Universidad Juan A. Maza, Lateral Sur del Acceso Este 2245, M5519 Guaymallén, Mendoza, Argentina

**Keywords:** Agriculture, Science, technology and society

## Abstract

The study of terroir, increasingly popular in scientific circles, remains a challenging field, particularly in terms of sensory analysis. This paper summarizes our own contribution to the field—an approach for tracing the typicity of wines by sensometrics, using the Malbec variety as a case study. This large-scale research fingerprinted 81 wines from 29 parcels from highly contrasting environments (varying climate, elevation, and soils), produced under standardized conditions in three consecutive vintages. Wines were evaluated through descriptive sensory analysis (DA) by a trained panel, and sensory descriptors were associated with different size geographic scales (zones, departments, and Geographic Indications (GIs)). The application of different sensometric tools allowed us to describe the typicity of wines and discriminate each region, proposing a novel methodology for the comprehensive evaluation of terroir from a sensory viewpoint. The vintage effect was very strong at the departmental and GI level, with aroma, taste and mouthfeel descriptors varying annually. However, certain origin descriptors remained consistent, providing insight into the typicity of Malbec. Considering the extension of the experimental study performed, this methodology provides a proof of concept for understanding both terroir and vintage effects from a sensorial perspective, offering wine producers and consumers a clear message backed by scientific evidence.

## Information

Wine is a complex matrix containing volatile and non-volatile components that interact with each other, and these interactions can affect the perception of aromas, flavor, and mouthfeel^1^. Therefore, sensory properties can significantly vary in wines with similar chemical characteristics. Chemical profiling is important for the discrimination of each region, but by involving the sensory perception of persons, it has a greater scope in the communication and interpretation of wines typicity, in this case, of the regions in which grapes are obtained. Also, the economic and cultural value of wine has, since antiquity, been closely tied to origin. Notions of quality and reputation were often evenly matched to the specific location of vineyards, their unique soil and climat—what would come to be known as terroir. The term is associated with the environmental conditions and cultural practices in the place where the grapes are grown—their direct influence on the chemical composition and sensory attributes, the character and quality of wines. The concept of terroir is an important communication vector in the wine industry. It has been defined as a cultivated ecosystem, involving such factors as the soil, topography, macro and microclimate of a particular viticultural site^2,3^. Because this ecosystem is cultivated, humans play a major role in the expression of terroir^4^. Cultural and socio-economic factors, therefore, as well as viticultural and oeonological techniques, are part of terroir^2^.

The belief that a particular product can only be produced within the confines of a specific terroir is one of the main conductive forces for the price of wines from certain areas^5^. Once the prerogative of Old World producers, today the term terroir is used by vintners and winemakers worldwide, while origin remains a moving factor in consumer choice^6^.

Few studies, however, have been able to support the typicity of wines in GIs, while others have struggled to recognize sensory markers specific to their location. Ballester at al^7^. suggested that future studies on the sensory impact of terroir should bypass the existing GIs system and build an experimental design based on objective and measurable terroir parameters, both chemical and sensory. In this vein, big scale studies over vintages should make it plausible to diminish the variability in categories or the lack of consensus, finding consistent sensory signatures for specific terroirs, supported by scientific evidence.

Two types of typicity have been defined for wines: a perceptual typicity—involving some type of sensory analysis, and a conceptual one: a mental representation subject to experiences as a social construct^8^. The sensory characteristics of the wines in each of the regions provide information on one kind of typicity: in this case, perceptual typicity. Previous studies have shown that geographic location has a direct influence on the chemical and sensory composition of wines in several countries and varieties^9–12^. But the identification of any consistent behavior for specific terroirs over different vintages has remained challenging, which could be the driving force to scientifically define the terroir.

Using the cv. Malbec as model, we developed an integrative approach for tracing the typicity of wines from different terroirs using sensory data. Malbec (*Vitis vinifera L*.) is a red grape variety that originated in France and is now widely cultivated in Argentina—accounting for 77% of Malbec acreage worldwide, thriving in a wide range of environments^13^. Data pertaining to its principal characteristics has been reported previously, including the observation that Mendoza exhibits a significant climate variability ranging from Winkler Region 5 to 1 in different zones, and that this variability is strongly influenced by altitude and proximity to the Andes Mountain range^14^. Mendoza has three main wine growing zones where approximately 82% of the province’s Malbec is grown: the East Zone, the First Zone and the Uco Valley. These three zones cover 6 departments (political divisions) and within them some Geographical Indications (GIs) have been declared. For the present study, wines from three large regions of Mendoza (zones), Argentina, including 6 departments and 12 GIs—a total of 81 independently elaborated wines over three vintages: 2016, 2017 and 2018 were analyzed by descriptive sensory analysis (DA).

DA is one of the few methodologies that can quantify an extensive range of sensory attributes for multiple products, proving itself an invaluable tool in wine sensory analysis. Previous studies have used DA to compare the behavior of wines from different regions of the world^15,16^. Different sensometric tools were proposed to discover the typicity of wines and discriminate each of the regions. The main goal was to develop a proof of concept for evaluating terroir from a sensory perspective to help wineries and consumers in processes of communication, recognition, and choice. Focusing on Malbec, we provided evidence of sensory descriptors and their association with origin for a range of geographic denominations: zones, departments, and GIs. Consistency and/or changes to descriptors of wine typicity across vintages were also investigated.

## Results and discussion

### Sensory attributes discriminate zones geographic denominations over vintages

**Fig. 1:**
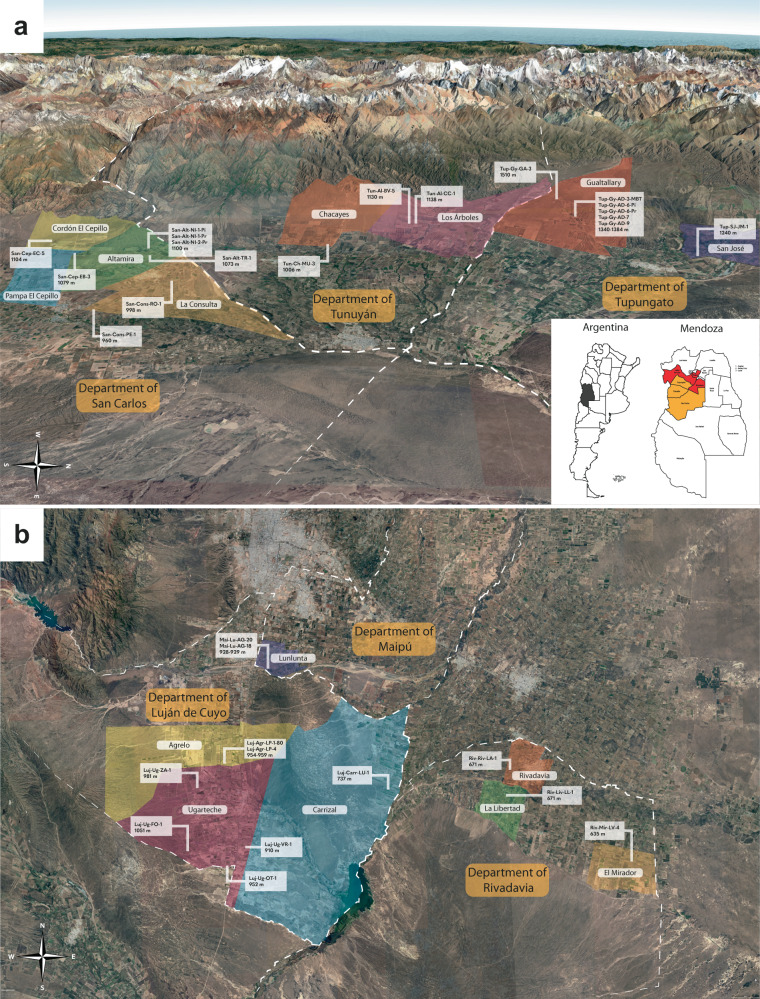
Satellite image of study parcels in Mendoza, Argentina. Satellite image of study parcels in the **a** Uco Valley, **b** First Zone, and East Zone of Mendoza, Argentina, with their respective GI and departments. Elevations are also shown.

From a wine production standpoint, Mendoza is divided into zones; the Uco Valley, for example, comprises the Departments of Tupungato, San Carlos, and Tunuyán (Fig. 1a). The main characteristics of the Uco Valley are a relatively cold climate with alluvial soils typical of the Andes mountains—where fluvial deposits predominate, generating fans that extend across the plains^17^. The First Zone encompasses the Departments of Maipú and Luján de Cuyo, where a warmer climate, in relation to the Uco Valley, predominates. As for the Eastern Zone, a single department—Rivadavia—was included in this study (Fig. 1b). Compared with the first two zones described, Rivadavia has the warmest climate, lower elevations, and predominantly wind-formed soils that are heavier, clay-based, and sandy.

For zones, the three-way MANOVA was significant for location, repetition, judge, location:judge interaction and repetition:judge interaction in vintages 2017 and 2018 (Supplementary table 1). Using a *p*-value < 0.05, the wines by zones were significant in 8 descriptors for 2017 vintage and 9 descriptors for 2018 vintage (Supplementary table 2).

For the 2017 vintage, significant descriptors were eucalyptus, hot (aroma), nutty, vegetable, smoky, bitter, astringency and hot (mouthfeel). For 2018 vintage, hot (aroma), red fruits, roses, smoky, humidity, acetic, astringency and spicy mouthfeel were significant. Hot (aroma), smoky and astringency were those with significant differences in both vintages (Supplementary table 2).

Vegetable, hot (mouthfeel), red fruits and astringency descriptors, have all been significant in previous studies on Malbec, where different zones and departments within Mendoza were compared^16,18^.

**Fig. 2:**
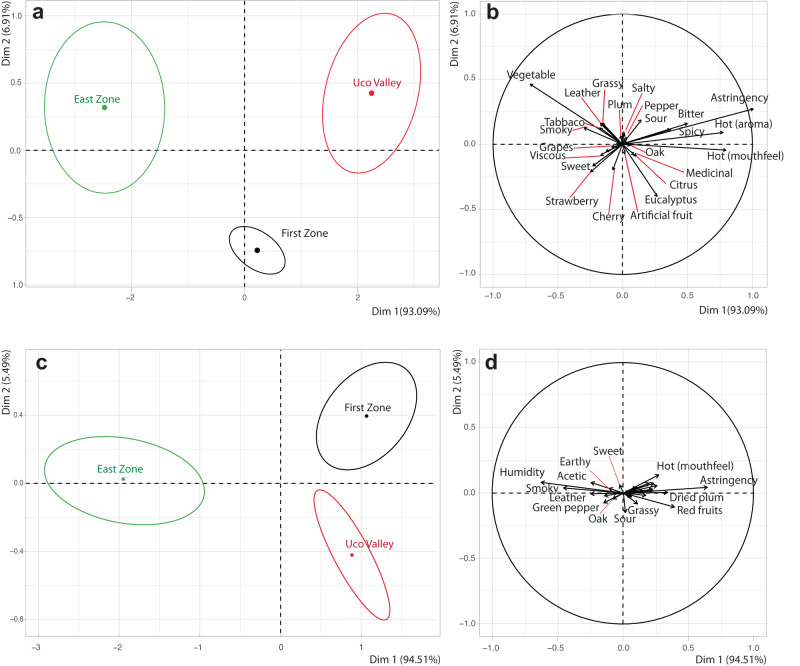
Principal components analysis with descriptive sensory data of Malbec wines vintage 2017 and 2018 evaluated by a trained panel using “Zones” as classification. **a** Confidence ellipses based on multivariate distribution of Hotelling’s test for *p* < 0.05 indicating 95% confidence intervals in vintage 2017. **b** Sensory attribute loadings in vintage 2017. **c** Confidence ellipses based on multivariate distribution of Hotelling’s test for *p* < 0.05 indicating 95% confidence intervals in vintage 2018. **d** Sensory attribute loadings in vintage 2018.

To better understand the differences between zones, a PCA was performed with all the descriptors involved, including those with no significant differences (Fig. 2). In 2017, a clear separation of each zone was observed (Fig. 2a). The East Zone was positively associated with vegetable, tobacco, smoky, sweet, viscous, leather, and grassy descriptors, while the Uco Valley was associated with eucalyptus, astringent, bitter, spicy, hot (aroma and mouthfeel) and sour. Eucalyptus and cherry appeared in the First Zone as well (Fig. 2b).

In 2018, the First Zone and Uco Valley were both positively associated with descriptors such as hot (mouthfeel), astringency, plum and red fruits, whereas the East Zone was associated with humidity, acetic acid, smoke, leather, green pepper and earthy aromas (Fig. 2c, d). PC2 contributes to the difference between First Zone and Uco Valley, where Uco Valley had descriptors associated with sour and grassy aroma.

In terms of vintage, there was a measure of consistency over zones: smoky and leather descriptors remained consistent in the East Zone across both vintages. Likewise, in the Uco Valley descriptors such as astringency and sour were present in both 2017 and 2018.

The Uco Valley has higher elevation conditions, thus higher UV-B exposure and lower temperatures which can affect the concentrations of phenolic compounds^19,20^, mainly those associated with descriptors such as astringency and bitterness, as reported previously^21,22^. The discrimination between zones observed in Fig. 2a and c is like our previously reported discrimination using chemical data for the same wines in the vintages: 2016, 2017 and 2018, where a strong influence of the vintage was also reported^14^.

Not all Mendoza wines are from very small specific places or parcels, with well-known terroir characteristics. Wine labels don’t always include specific information about the origin—the use of such general terms as: Uco Valley, First or East Zone is common. Data from this study provides sensory typicity information for these regions, contributing to the identification of their unique characteristics, and providing a tool for assessing a wine’s origin within a regional geographic denomination. Beyond the identification of GIs or parcels capable of providing highly recognized wines, it is both meaningful and useful to ascertain the sensory characteristics that make the wines of a zone unique—with aim at protecting their typicity and traceability. Both the physicochemical and sensorial data are important from scientific and commercial points of view, building regional identity from individual parcels to zones. In this vein, the evidence presented here offers new knowledge to trace the typicity of Argentinean Malbec and to build a representative sensory typology of these wines. From a commercial standpoint, our research provides scientific evidence to support new world winemaking regions in understanding the typicity of their wines.

**Fig. 3:**
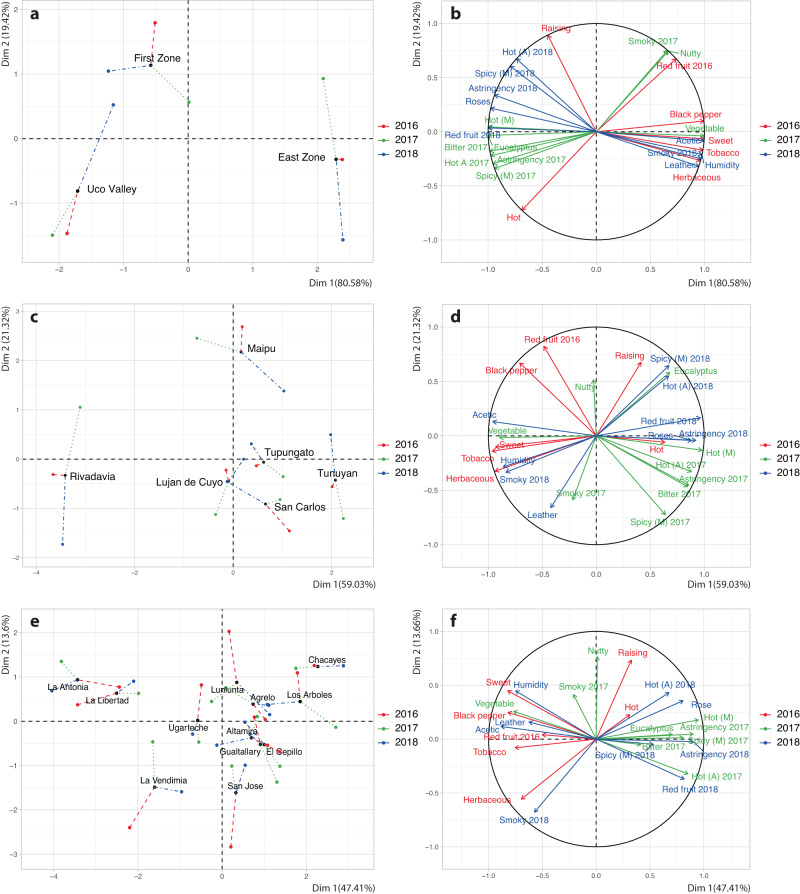
Multifactor analysis using different scales of geographical denominations for the discrimination. Zones (**a**, **b**), Departments (**c**, **d**) and GIs (**e**, **f**) were used as classification. In the plots consensus MFA sample space (**a**, **c**, **e**), the length of the line is inversely related to the strength of the agreement. Data from 2016 vintage was obtained from our previous publication (Urvieta et al.^18^).

To understand the discrimination of the zones by sensory data over different vintages, an MFA analysis integrated the data obtained from each zone over the 3 years examined. For the 2016 vintage, data from Urvieta et al. was included^18^. As Fig. 3a shows the influence of vintage in zones classification is clear. The Uco Valley and East Zone were best explained by the second dimension. There was a better agreement between the First zone than in the other two zones. Sensory descriptors that were loaded on the MFA (Fig. 3b) in proximity and agreement between the three vintages were hot aroma, vegetable/herbaceous, astringency and smoky. The aroma of red fruits was associated with the East Zone in the 2016 vintage, but with the regions of the Uco Valley and the First zone in 2018. The vintage effect has a very strong impact on the chemical composition of Malbec wines from Mendoza, and therefore on its sensory properties. In terms of climatic conditions and unlike the 2017 and 2018 vintages, the 2016 vintage was classified as very cold, rainy and humid, conditions that are not typical for Mendoza. In the case of the 2017 and 2018 vintages, the climatic characteristics were similar to those historically observed in Mendoza, and this was reflected in the chemical characteristics of the wines, which shared some of the discriminant descriptors. The 2017 and 2018 vintages showed climatic characteristics similar to Mendoza’s historical averages, and the vintages can be easily predicted using models such as PCA and PLS-DA^14^.

### Sensometrics partially discriminates departments and evidence a strong vintage effect

The same number of significant descriptors was found when ANOVA was performed using departments as geographic classification denominations (Supplementary tables 3 and 4). Significant descriptors for the 2017 vintage were eucalyptus, hot aroma, nutty, vegetable, smoky, bitter, astringency and hot (mouthfeel). For 2018, descriptors with significant differences were hot aroma, leather, red fruits, roses, smoky, humidity, acetic, astringency and spicy. Descriptors that are also significant in both vintages and within this particular zonal classification are hot aroma, smoky, astringency and spicy (Supplementary Tables 1 and 2). In our previous study on 2016 vintage wines using the same department classification, the descriptors with significant differences were red fruits, raisins, black pepper, herbaceous, tobacco, hot and sweet^18^. The astringency descriptor was significant in the 2017 and 2018 vintages (Supplementary tables 4 and 6), but not in 2016^18^. According to data from these same years, wines from the 2017 and 2018 vintages had an increased level of anthocyanins and flavonols. Descriptors such as astringency and bitterness are associated with these compounds and are relevant in the discrimination of departments for these vintages.

**Fig. 4:**
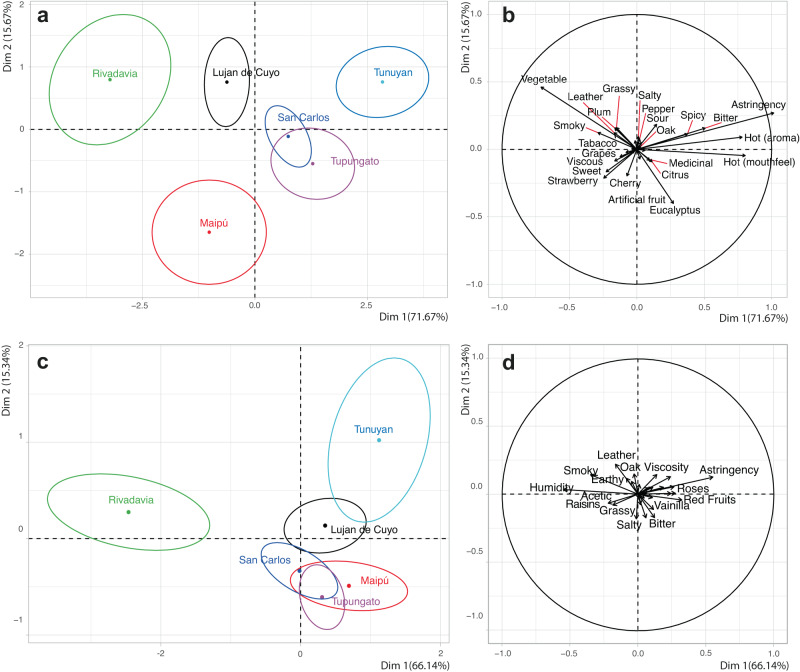
Principal components analysis with descriptive sensory data of Malbec wines vintage 2017 and 2018 evaluated by a trained panel using “departments” as classification. **a** Confidence ellipses based on multivariate distribution of Hotelling’s test for *p* < 0.05 indicating 95% confidence intervals in vintage 2017. **b** Sensory attribute loadings in vintage 2017. **c** Confidence ellipses based on multivariate distribution of Hotelling’s test for *p* < 0.05 indicating 95% confidence intervals in vintage 2018. **d** Sensory attribute loadings in vintage 2018.

When applying the same sensometric tools for the department classification, the aggregate effect and the consistency in discrimination is different from the classification by zones. We observed that while some departments have a clear separation, others cannot be easily discriminated against (Fig. 4). Since the objective of this study was to evaluate the typicity, all the aromas and flavors that each region has in common were included independently if they were significant or not in the sensory analysis. Figure 3b and d show the descriptors associated with each department.

In both vintages it is consistent that Rivadavia is separated from the rest of the departments, mainly with descriptors associated with vegetal, smoky, grassy, leather, plum and humidity. The same result in wines from Rivadavia was observed for the 2016 vintage, where it was associated with herbaceous, tobacco, black pepper and sweet^18^. Of the descriptors mentioned, herbaceous or its family of aromas is the most consistent in all vintages for this department. Aromas within the vegetal family have been reported in previous studies of Malbec—descriptors such as cooked vegetal, green or vegetal characters, including herbal or herby^16,23^. As mentioned above and based on climatic data published in other studies, Rivadavia lies in the warmest region of Mendoza at a lower altitude (650 m asl) than Valle de Uco and the First Zone regions.

The astringency was associated with departments of the Uco Valley (Fig. 4). This descriptor is closely linked to the concentration of phenolic compounds. The concentration of phenolic compounds, responsible for flavors and tactile sensations, is influenced by environmental conditions—temperature, rainfall, light intensity, soil—where the vineyards are located^24,25^. Previous studies showed that as elevation increases, the intensity of ultraviolet-B (UV-B) radiation also increases due to the reduced absorption of atmospheric gases at higher altitudes, a phenomenon further associated with an increase in phenolic compounds such as quercetin, *trans-*resveratrol and di-hydroxylated anthocyanins^19,20^. These environmental characteristics of higher elevation and cooler climates are those found in the departments (and consequently GIs) in the Uco Valley zone studied. The information presented suggested a potential correlation between chemical and sensory data because some of these compounds, such as quercetin and (+)-catechin, have been associated with the astringency and bitterness descriptors of wines.

The MFA with the averages of each sensory variable for the three harvests classified by department showed a clear influence of vintage in some departments of Mendoza, such as Rivadavia and Maipú (Fig. 3c, d). Rivadavia is more strongly correlated with the second dimension, while the observation from Maipú is more strongly correlated with the first dimension. The worst consensus on the discrimination of Rivadavia and Maipú is in the 2017 and 2018 vintages. San Carlos and Tunuyán have a good consensus in the 3 years of study. Tupungato, Luján de Cuyo and San Carlos could be potentially forming a large group due to similar sensory characteristics. Looking at the individual PCAs of the 2017 and 2018 vintages (Fig. 4a, c), and the 2016 data from our previous paper, they could be closely related by their sensory characteristics^18^. The RV coefficient calculated between the 2016 and 2017 vintage configurations is 0.89, 2016 and 2018 is 0.86 and 2017 and 2018 is 0.71.

### Understanding the interactions between sensory typicity and geographic proximity of GIs across vintages

A MANOVA analysis was performed using sensory data by GIs classifications. Using a *p*-value < 0.05, the wines by GI were significant in 12 descriptors for vintage 2017 and in 5 descriptors for vintage 2018 (Supplementary Tables 5 and 6).

**Fig. 5:**
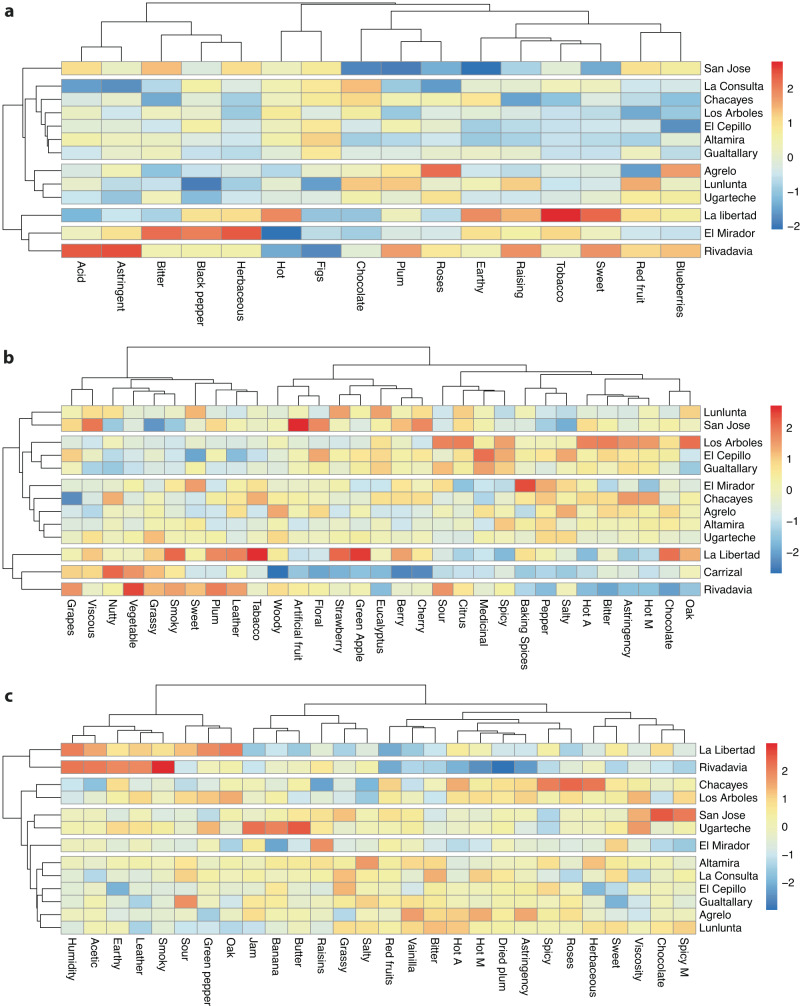
Heatmaps and hierarchical clustering of GIs (small areas) by sensory descriptors in Malbec wines over different vintages. 2016 (**a**), 2017 (**b**) and 2018 (**c**). Values and cell colors indicate a relative intensity for each GI; blue corresponds to the minimum value and red to the maximum value (‘heatmap colors’).

Figure 5 shows heatmaps with the GI groupings when all the sensory variables were used over 3 vintages, including the 2016 vintage with data from Urvieta et al. ^18^. These groupings help to understand the differences and similarities between GIs using an unsupervised analysis. Most of the GIs in all parts of the world, as in the case of Argentina, were determined based on political boundaries. These limits were not decided based on the sensory differences of the wines or their typicity. There are cases such as Chacayes and Los Arboles that form a group in the 2018 vintage. In terms of location and geographic proximity the grouping is logical because they belong to the same alluvial cone and are located next to each other^17^. The fact that they form a cluster does not mean that the wines are identical, only that they share sensory descriptors and characteristics that group them by similarities, just as the combination of descriptors makes the wines from these sites unique.

The clustering observed in this study with many GIs using sensory data represents a novel finding that has not been previously reported in the literature. This result provides a valuable contribution to the field of terroir research, as it allows for a better understanding of how GIs can be created or reclassified based on sensory characteristics. Moreover, our study facilitates the identification of the unique attributes that define each region, thereby contributing to the conceptual typicity and improving communication within the wine industry. Overall, our findings have important implications for future research in this area and demonstrate the usefulness of using sensory data to study GIs.

The multiple factor analysis (MFA) (Fig. 3), integrating sensory variables for the three vintages using GIs as classification criteria, presented a less clear distribution of the GIs by geographic location. However, there are places that are separated from the rest that are also geographically close. La Libertad, La Antonia and La Vendimia—all located on the left side of the MFA—were associated with red fruit, vegetable, herbaceous, leather, humidity, black pepper, smoky, acetic and sweet. This separation of GIs from the zone was also observed in the classification by region and departments, showing consistency in the classification over different geographic denominations.

Los Arboles and Chacayes are in the upper right quadrant. Both GIs are in the same department and next to each other. A good consensus is observed across all three vintages for these two GIs.

MFA analysis is useful in understanding that there are zones where the impact of the vintage was stronger than in others. Understanding the behavior of each zone throughout different vintages is crucial, the more when vintages have contrasting climates. So, for example, the 2016 and 2018 vintages were very different in terms of climate, causing GIs such as Lunlunta to present a different behavior for each vintage, unlike other GIs such as Agrelo, where the consensus between all three vintages is much greater, probably evidencing more consistency of sensory characteristics of that place between vintages. Although climate is a fundamental element of terroir, recent variations in ‘typical’ climatic patterns could challenge the traditional dimensions of wine typicity from specific locations. Given that climatic conditions are increasingly dynamic and subject to rapid, extreme changes, the sensory profiles of wines may start exhibiting ‘atypical’ characteristics, as observed in our study with the 2016 vintage. This trend highlights the potentiality of the proof of concept presented here to understand the typicity of a grape variety, in this case Malbec, but readily translatable to other grape varieties, in wines from regions around the world.

Tracing the typicity of wines from different origins has implications for both consumers—deepening their understanding of the product—and producers—furthering the construction of robust signatures to support the concept of terroir with scientific evidence. From a sensory analysis perspective, the possibilities presented here: different ways of classifying the typicity of wines at varying geographic scales and across vintages, may move the discussion towards greater consensus, by providing evidence of consistent sensory signatures for specific terroirs. Looking more specifically at the Malbec findings, knowing the typicity of Malbec and whether it is possible to distinguish between different geographic scales allows for a more detailed and confident communication of the identity and characteristics of wines from Argentina. The presence of common sensory descriptors in different vintages, affected by different climatic characteristics, allows for wines from the different geographical areas under examination to be fingerprinted for typicity. In the future, we believe the methodology presented here could contribute to an extensive understanding of the effect of terroir from a sensory perspective.

## Methods

### Vineyard sites and winemaking

Mendoza has three large wine zones where ~82% of the province’s Malbec is grown: the East Zone, First Zone and Uco Valley. These three zones cover 6 Departments (political divisions of the province), and inside them some GIs have been declared. Figure 1a shows the location of the parcels and GIs in the Uco Valley, which includes the Departments of San Carlos, Tunuyán and Tupungato. Figure 1b shows the location of the parcels in the First Zone, which includes the Departments of Luján de Cuyo and Maipú. The Department of Rivadavia, which belongs to the East Zone, is also included in Fig. 1b.

Grapes from the 29 parcels were elaborated out at the Catena Institute of Wine’s experimental winery located in Agrelo, Luján de Cuyo, Mendoza. This was realized in triplicate for each parcel, except for three parcels, done in duplicate due to the small area of these parcels. Table 1 provides information for each parcel, including elevation and harvest dates. The Luj-Ug-OT-1, Luj-Ug-VR-1 and Luj-Carr-LU-1 parcels were only harvested in 2017. The San-Cons-RO-1 parcel was only harvested in 2018. All other parcels were included in both vintages. The grapes were first destemmed, then crushed, and the resultant must was transferred into 800 L plastic vessels for fermentation. At the onset of incubation, 50 mg/L of SO_2_ (Enartis América Latina, Mendoza, Argentina) were added. After 24 h, 20 g/hL of Lavin EC-1118 active dry yeast (Lallemand Inc., Montréal, QC, Canada) were inoculated into the fermentation vessels. One day after inoculation, 100 mg/L of (NH_4_)_3_PO_4_ were added as a nitrogen source for the yeast. The fermentation temperature was maintained at 25 ± 2 °C, with density (°Brix) and temperature monitored every 12 h. Following the alcoholic fermentation and a maceration period of 10 days, 50 L of the drained wine were removed from each vessel. Wine presses were not utilized. After 5 days of aging in stainless steel tanks, 1 g/L of selected Lavin VP41 bacteria (Lallemand Inc., Montréal, QC, Canada) was introduced for malolactic fermentation. This stage was deemed complete when the malic acid content reduced to below 0.2 g/L, as determined using OenoFoss (FOSS Analytical A/S, Hillerød, Denmark). pH monitoring of the wines was conducted throughout and upon the completion of malolactic fermentation. Due to significant variability in the acidity conditions of the wines, adjustments were made with tartaric acid to maintain a pH <3.79. This strategy enhances microbiological control and reduces risks associated with Brettanomyces and acetic bacteria. Thereby, avoiding sensory deviations that could interfere with the sensory characteristics of the wines given by the origin of the grapes. Similar criteria was used in other terroir studies^26^. For grapes from warmer areas included in this study, adjustments included the addition of up to 2.1 g/L of tartaric acid in some cases. Although acidity adjustments can alter sensory aspects such as taste and mouthfeel sensations, these modifications closely represent enological practices and may not alter the aroma profile^27^. After malolactic fermentation finished, decantation was carried out to remove lees. Subsequently, SO_2_ in the form of K_2_S_2_O_5_ (Laffort Oenologie, France) was added to achieve a final concentration of 35 mg/L of free SO_2_. The wines were stored for 3 months in 50 L stainless steel tanks at 13–15 °C. The Malbec wines were bottled under nitrogen, in 750 mL dark glass bottles with tin screw caps—selected over natural cork as stoppers to prevent any potential trichloroanisole contamination and/or variable oxygen incorporation.

**Table 1:** Mendoza Malbec parcels site information

Locations										
Zones	Departments	GIs	Parcels	Planting year	Vineyard Orientation	Latitude	Longitude	Elevation (m)	2017	2018
First Zone	Lujan de Cuyo	Agrelo	Luj-Agr-LP-1-80	2006	N–S	33° 9′58.02″S	68°54′53.35″W	959	6-mar	12-mar
			Luj-Agr-LP-4	1996	N–S	33° 9′59.04″S	68°54′27.51″W	954	8-mar	12-mar
		Ugarteche	Luj-Ug-FO-1	2008	N–S	33°16′11.98″S	68°58′28.41″W	1051	16-mar	26-mar
			Luj-Ug-OT-1	2000	N–S	33°17′26.0″S	68°55′11..3″W	952	17-mar	
			Luj-Ug-ZA-1	2001	N–S	33°11′38.22″S	68°57′21.67″W	981	31-mar	28-mar
			Luj-Ug-VR-1	2011	N–S	33°16′6.46″S	68°53′23.33″W	910	2-mar	
		Carrizal	Luj-Carr-LU-1	2008	*	33°12′21.56″S	68°40′38.20″W	737	8-mar	
	Maipu	Lunlunta	Mai-Lu-AG-18	1922	N–S	33° 2′58.31″S	68°50′54.22″W	928	14-mar	20-mar
			Mai-Lu-AG-20	1922	N–S	33° 3′6.35″S	68°50′38.90″W	929	15-mar	21-mar
East Zone	Rivadavia	El Mirador	Riv-Mir-LV-4	2001	*	33°18′30.24″S	68°19′25.15″W	635	8-mar	21-mar
		La Libertad	Riv-Lib-LL-1	1921	N–S	33°13′15.03″S	68°30′10.64″W	671	14-mar	22-mar
		Rivadavia	Riv-Riv-LA-1	2003	N–S	33°11′19.88″S	68°29′48.59″W	671	10-mar	22-mar
Uco Valley	San Carlos	La Consulta	San-Cons-RO-1	1960	N–S	33°42′53.25″S	69° 6′43.52″W	998		6-abr
		Altamira	San-Alt-TR-1	2005	N–S	33°4′48.43″S	69° 9′16.60″W	1073	24-mar	4-mar
			San-Alt-NI-1-Pi	2000	N–S	33°45′22.92″S	69°10′40.91″W	1100	22-mar	12-mar
			San-Alt-NI-1-Pr	2000	N–S	33°45′25.96″S	69°10′34.54″W	1100	23-mar	12-mar
			San-Alt-NI-2-Pr	2000	N–S	33°45′20.10″S	69°10′38.89″W	1100	24-mar	14-mar
		Pampa El Cepillo	San-Cep-EB-3	2005	N–S	33°48′39.39″S	69° 10′7.95″W	1079	17-mar	16-mar
			San-Cep-EC-5	2010	N–S	33°50′21.35″S	69°11′44.80″W	1104	20-mar	12-mar
	Tunuyán	Chacayes	Tun-Ch-MU-3	2005	N–S	33°36′46.67″S	69°11′39.55″W	1006	22-mar	29-mar
		Los Arboles	Tun-Al-BV-5	2005	N–S	33°32′37.22″S	69°14′29.37″W	1130	22-mar	22-mar
			Tun-Al-CC-1	2002	N–S	33°32′16.33″S	69°14′45.98″W	1138	22-mar	22-mar
	Tupungato	Gualtallary	Tup-Gy-AD-3-MBT	1998	N–S	33°23′56.47″S	69°15′34.29″W	1384	27-mar	23-mar
			Tup-Gy-AD-6-Pi	1999	N–S	33°23′37.26″S	69°14′59.06″W	1349	13-mar	14-mar
			Tup-Gy-AD-6-Pr	1999	N–S	33°23′42.29″S	69°14′58.62″W	1350	28-mar	22-mar
			Tup-Gy-AD-7	1999	N–S	33°2′48.65″S	69°14′57.03″W	1346	24-mar	13-mar
			Tup-Gy-AD-9	1999	N–S	33°23′43.28″S	69°14′50.31″W	1340	24-mar	22-mar
			Tup-Gy-GA-3	2011	N–S	33°24′3.40″S	69°18′7.25″W	1510	3-abr	28-mar
		San José	Tup-SJ-JM-1	2007	N–S	33°18′59.23″S	69°10′5.37″W	1240	22-mar	27-mar

^*^Overhead trellis.

### Sensory analysis

The sensory profiles of the Mendoza Malbec wines were evaluated ~6 months after bottling, in two descriptive DA sessions performed in the wine sensory laboratory of the University of California, Davis, and at the INTA’s sensory laboratory in Mendoza, Argentina. So, Malbec wines from the 2017 vintage were evaluated in October 2017, and those from the 2018 vintage were evaluated in October 2018. A fermentation replicate of each viticultural site or parcel was randomly selected and used for descriptive sensory analysis—including 28 wines for the 2017 vintage and 26 wines for 2018. Both sensory panels were conducted using the generic descriptive sensory analysis methodology described by Lawless & Heymann, 2010, which consists of selection of panelists, generation of attributes by consensus, concept formation, scoring and quantification of each descriptor in the wines^28^.

Panelists were recruited through advertising within the University. For the 2017 vintage wines, a total of 10 panelists (7 women) participated, ranging in age from 21–55 years. For the 2018 vintage wines, 14 panelists (9 women) were recruited, aged 24–59 years, many with prior experience in wine descriptive analysis. Lasting ~9 h, training sessions for both panels consisted of an introduction to sensory analysis in wines, attribute generation, discussion, consensus on reference standards, and practice in the use of attribute intensity scales. The sensory panel 2017 conducted at UCDavis was reviewed and approved by an Institutional Review Board (“IRB”). The 2018 sensory panel, carried out at INTA, was approved by members of the INTA sensory analysis laboratory, Mendoza, Argentina experimental station.

Descriptive analysis panels for the 2017 and 2018 vintages rated 30 and 28 attributes, respectively. Since descriptive analysis is a consensus technique, the descriptors evaluated by each panel are different for each vintage. Supplementary tables 7 and 8 show the descriptors and references used for each year.

Panelists evaluated 28 (2017) and 26 (2018) parcels of Malbec wines in triplicate over 15 sessions—equivalent to 6–7 wines per session presented in a randomized block design. Before each testing session, reference standards were available to refresh memory.

Each evaluation session was carried out in individual sensory booths. Each sample had 30 ml of wine at a temperature of 20 °C in black tasting glasses (ISO 3591–1977), covered with plastic lids. Each sample was coded with three-digit random numbers. For each of the descriptors, panelists rated intensity on an unstructured linear scale anchored with the terms “low” and “high” at each end of the scale. A 60-second break between each sample was included, where the panelist cleaned the palate with water and salt-free crackers.

The order of presentation of the samples was randomized using a modified Williams Latin Square design. The 2017 panel data was collected on FIZZ software (ver. 2.51 G; Biosystèmes, Couternon, France) and SOLDESA software^29^ was used for the 2018 panel.

In the case of the 2016 harvest data, these were sourced from Urvieta et al.^18^. This dataset was generated using a consistent methodology, facilitating the analysis of vintage effects through MFA.

### Data analysis

Data was analyzed using the software platform R 3.2.2^30^. MANOVA analysis was performed using three-way MANOVA (judge, locations, rep and all two-way interactions) on all attributes. Three-way ANOVA with two-way interactions to analyze the descriptor’s attributes with the missing values. The missing values (2.85% in 2017 and 4.27% in 2018) were inputted with the “missMDA” package in R^31^. In two sections of the results, principal component analysis (PCA) with a covariance matrix was applied on the wine sensory data, including panelists at two levels of locations, zones, and departments. To understand terroir and typicity, the criteria used in the PCA, cluster analysis and heatmaps, was to include all the variables in such analysis. In this way, it was possible to describe the complete range of sensory variables of each zone, department and GIs, and not only discriminate between them. Confidence ellipses in the PCA indicating 95% confidence intervals were based on the multivariate distribution of the Hotelling’s test for *p* < 0.05 and were constructed using SensoMineR panellipse function on R^32^. To understand grouping and differences between GIs, cluster analysis was constructed using the Euclidean and Ward.D2 clustering technique and heatmap was performed with the ‘pheatmap’ package. The MFA was performed using the Factoextra package utilizing significant variables. Data published in Urvieta et al. was incorporated into the MFA analysis to integrate 3 years of studies for zones, departments, and GIs classification^18^.

### Reporting summary

Further information on research design is available in the Nature Research Reporting Summary linked to this article.

### Supplementary information

Supplementary information

Reporting summary

## Data Availability

The datasets generated during and/or analysed during the current study are available from the corresponding author.
