# Supplementary material for: Tracing the origin of Argentine Malbec wines by sensometrics
Source: NPJ Sci Food. 2024 Feb 21;8:14. doi: 10.1038/s41538-024-00252-3 (PMC10879186; doi:10.1038/s41538-024-00252-3)
Supplement: Supplementary file 1 — Supplementary information [file 41538_2024_252_MOESM1_ESM.pdf]

Supplementary information of the article Tracing the Origin of Argentine Malbec Wines by Sensometrics

Roy Urvieta, Hildegarde Heymann, Annegret Cantu, Aníbal Catania, Fernando Buscema, Rubén Bottini, Ariel Fontana

Supplementary table 1. MANOVA three way by zones of vintage 2017 and 2018

	Vintage 2017	Vintage 2018
Zones	2.20E-16	4.44E-13
Repetition	2.20E-16	2.20E-16
Judge	2.20E-16	2.20E-16
Zones:repetition	0.07871	0.5419
Zones: Judge	6.29E-14	1.22E-09
Zones: Judge	2.20E-16	2.20E-16

Supplementary table 2. ANOVA with pseudomixed model with sensory descriptors of Malbec wines 2017 and 2018 by zones.

Vintage 2017				Vintage 2018			
Aroma descriptors	p-valor	Taste and mouthfeel descriptors	p-valor	*Aroma descriptors*	p-valor	Taste and mouthfeel descriptors	p-valor
Berry	0.496	*Sweet*	0.521	*Vanilla*	0.228	*Sweet*	0.615
Strawberry	0.445	*Sour*	0.0995	*Banana*	0.14	*Bitter*	0.237
Chocolate	0.934	*Bitter*	**0.0135**	*Spicy*	0.517	*Sour*	0.152
Citrus	0.235	*Salty*	0.945	*Jam*	0.408	*Salty*	0.676
Plum	0.388	*Astringency*	**0.000344**	*Chocolate*	0.818	*Astringency*	**0.000167**
Eucalyptus	**0.0098**	*Viscosity*	0.374	*Green pepper*	0.314	*Spicy*	**0.0444**
Grassy	0.195	*Hot*	**0.000647**	*Dried plum*	0.416	*Viscosity*	0.624
Cherry	0.828	*Spicy*	0.0701	*Earthy*	0.262	*Hot*	0.0557
Grapes	0.833			*Hot*	**0.025**		
Hot	**0.000219**			*Grassy*	0.255		
Nutty	**0.0334**			*Herbaceous*	0.666		
Artificial fruit	0.888			*Leather*	**0.0365**		
Baking Spices	0.47			*Red fruits*	**0.00067**		
Pepper	0.969			*Roses*	**0.0225**		
Vegetables	**0.000164**			*Raisins*	0.65		
Oak	0.888			*Smoky*	**0.0133**		
Woody	0.83			*Humidity*	**0.0108**		
Smoky	**0.0357**			*Acetic*	**0.0294**		
Green Apple	0.723			*Butter*	0.29		

Leather	0.235	*Oak*	0.594
Medicinal	0.189		
Tobacco	0.063		
Floral	0.607		

Supplementary table 3. MANOVA three ways by departments of wines 2017 and 2018

	Vintage 2017	Vintage 2018
Departments	2.20E-16	2.20E-16
Repetition	2.20E-16	2.20E-16
Judge	2.20E-16	2.20E-16
Departments:Repetition	0.03966	0.383
Departments: Judge	1.11E-12	2.20E-16
Repetition: Judge	2.20E-16	2.20E-16

Supplementary table 4. ANOVA with pseudomixed model with sensory descriptors of Malbec wines 2017 and 2018 by departments

Vintage 2017				Vintage 2018			
Aroma descriptors	p-value	*Taste and mouthfeel descriptors*	p-value	*Aroma descriptors*	p-value	Taste and mouthfeel descriptors	p-value
Berry	0.513	*Sweet*	0.408	*Vainilla*	0.289	*Sweet*	0.188
Strawberry	0.174	*Sour*	0.0833	*Banana*	0.162	*Bitter*	0.319
Chocolate	0.968	*Bitter*	**0.0106**	*Spicy*	0.81	*Sour*	0.248
Citrus	0.32	*Salty*	0.886	*Jam*	0.0812	*Salty*	0.112
Plum	0.65	*Astringency*	**9.79E-07**	*Chocolate*	0.827	*Astringency*	1.64E-05
Eucalyptus	**0.000628**	*Viscosity*	0.653	*Green pepper*	0.585	*Spicy*	0.23
Grassy	0.174	*Hot*	**0.000114**	*Dried plum*	0.134	*Viscosity*	0.246
Cherry	0.505	*Spicy*	**0.0421**	*Earthy*	0.46	*Hot*	0.0807
Grapes	0.748			*Hot*	0.0923		
Hot	**1.75E-06**			*Grassy*	0.106		
Nutty	**0.0115**			*Herbaceous*	0.844		
Artificial fruit	0.298			*Leather*	0.00305		
Baking Spices	0.564			*Red fruits*	0.0624		
Pepper	0.554			*Roses*	0.0869		
Vegetables	**1.64E-06**			*Raisins*	0.427		
Oak	0.202			*Smoky*	0.00188		
Woody	0.981			*Humidity*	0.02216		
Smoky	**0.0228**			*Acetic*	0.00573		

Green Apple	0.812	*Butter*	0.0534
Leather	0.316	*Oak*	0.436
Medicinal	0.201		
Tobacco	0.0596		
Floral	0.657		

Supplementary table 5. MANOVA three way by GIs of wines 2017 and 2018

	**Vintage 2017**	**Vintage 2018**
GIs	2.20E-16	2.20E-16
Repetition	2.20E-16	2.20E-16
Judge	2.20E-16	2.20E-16
GIs:Repetition	0.3584	0.6459
GIs:Judge	3.16E-13	2.20E-16
Repetition: Judge	2.20E-16	2.20E-16

Supplementary table 6. ANOVA with pseudomixed model with sensory descriptors of Malbec wines 2017 and 2018 by GIs

**Vintage 2017**				**Vintage 2018**			
Aroma descriptors	p-valor	Taste and mouthfeel descriptors	p-valor	*Aroma descriptors*	p-valor	Taste and mouthfeel descriptors	p-valor
Berry	0.567	*Sweet*	0.287	*Vainilla*	0.234	*Sweet*	0.121
Strawberry	0.0786	*Sour*	**0.0332**	*Banana*	0.316	*Bitter*	0.685
Chocolate	0.967	*Bitter*	**0.042**	*Spicy*	0.749	*Sour*	0.193
Citrus	0.523	*Salty*	0.901	*Jam*	0.109	*Salty*	0.129
Plum	0.423	*Astringency*	**1.40E-07**	*Chocolate*	0.9	*Astringency*	**2.24E-09**
Eucalyptus	**0.000211**	*Viscosity*	0.609	*Green pepper*	0.291	*Spicy*	0.0936
Grassy	0.279	*Hot*	**5.33E-05**	*Dried plum*	0.103	*Viscosity*	**0.0113**
Cherry	0.163	*Spicy*	**0.0327**	*Earthy*	0.253	*Hot*	**0.00222**
Grapes	0.803			*Hot*	0.0562		
Hot	**3.01E-07**			*Grassy*	0.425		
Nutty	**0.0447**			*Herbaceous*	0.75		
Artificial fruit	0.23			*Leather*	0.0509		
Baking Spices	0.408			*Red fruits*	0.103		
Pepper	0.757			*Roses*	0.0626		
Vegetables	**1.32E-11**			*Raisins*	0.164		
Oak	0.395			*Smoky*	**4.88E-05**		
Woody	0.995			*Humidity*	**0.00138**		

Smoky	**0.0447**	*Acetic*	0.0509
Green Apple	0.514	*Butter*	0.742
Leather	0.573	*Oak*	0.739
Medicinal	0.119		
Tobacco	**0.0295**		
Floral	0.42		

Supplementary table 7. Attributes used in descriptive sensory analyses (DA) to rate the sensory profiles of Malbec wines from 2017 vintage and the reference standards used.

Attribute	**Reference standard**
Aroma	
Berry	3 blueberries + 3 g blackberry in small pieces + 3 g raspberry in small pieces / Blueberries of Best Byet frozen mixed berry medley (Distributed by C & S wholesale Grocers, INC). Frozen blackberries of Best Byet (Product of Mexico, Distributed by C & S wholesale Grocers, INC). Best Byst frozen raspberries (Product of Serbia, Distributed by C & S Wholesale Grocers, INC).
Strawberry	10 g fresh strawberry in small pieces / Frozen whole strawberries of Best Byst (Product of Mexico, Distributed by C & S wholesale Grocers, INC).
Chocolate	4g 75% shaved dark chocolate / Artisana Organics Venezuelan Cacao criollo (Product of Venezuela, Distributed by Premier Organics, Oakland, CA)
Citrus	Grapefruit pink oil 4 drops + lemon oil 2 drops + 15 mL H2O / Grapefruit pink 100% pure essential oil of citrus paradisi (Manufactured for Nugget Markets, Woodland, CA). 100% pure lemon essential oil of citrus limonum (Manufactured for Nugget Markets, Woodland, CA).
Plum	1/2 Prune Pitted / Sunsweet Amazin prunes pitted (Product of USA).
Eucalyptus	10 ml (Solution of 4 drops of eucalyptus oil in 200 ml of H20) + 40 ml of base wine / Aura Cacia pure essential oil Eucalyptus radiata (Distributed by Frontier Natural Products CO-OP). Base wine Franzia Merlot (Import by Franzia vineyard, Ripon, CA. Product of Chile)
Grassy	2 g of fresh grass in small pieces
Cherry	10 gr of dark sweet cherries in small pieces / Dole (Westlake Village, CA)
Grapes	8 gr fresh table grapes + 1 mL Concord Grape Juice / Red seedless table grapes Pretty lady (Delano, CA). Juice Kedem (Produced by Kedem Food Products. Marlboro, NY)
Hot	25 ml 15% V / V Vodka (9 ml + 16 ml of H2O) / Seagram extra smooth Vodka (Lawrenceburg, IN)
Nutty	2 almonds + 1 g walnut + 2 unsalted peanuts / raw almonds, walnut Trader Joe's (Product of USA). Raw california walnut baking pieces Trader Joe's (Product of USA). Roasted unsalted peanuts Trader Joe's (Product of USA).
Artificial fruit	40 mL Vitamin water XXX acai-blueberry-pomegranate.
Baking Spices	0.1 g of ground cinnamon + 1 clove + 0.3 g of nutmeg / ground cinnamon Trader Joe's (Monrovia, CA). Whole cloves McCormick & CO, INC (Packed in USA). Ground Nutmeg McCormick & CO, INC (Packed in USA).
Pepper	1/4 tsp coarse ground black pepper + 40 ml of water / Kirkland Signature (Distributed by Costco Wholescale Corporation)
Vegetables	1.5 g canned asparagus + 1.5 g canned green beans

Oak	35 ml French Oak M+T (15 g / 400 ml by 4 hs) / Innerstave (Sonoma, CA).
Woody	5.5 gr of chip trees + 1 piece of cedar wood + 20 mL of water / Cedar balls (Cedar Fresh, product of USA)
Smoky	4 drop Liquid smoke in 50 mL H2O / Colguin (Dallas, TX)
Green Apple	10 gr of green apple in small pieces / Daisy Girl organics (Product of USA)
Leather	6 piece of 3 cm leather tan + 5 ml of water / Kiwi outdoor Johnson company
Medicinal	5 mL cough syrup cherry flavor / Triaminic Syrup (Distributed by GSK Consumer Healthcare, Warren, NJ)
Tobacco	0.1 gr of tobacco / Stave-Aged Virginia 35 Ribbon pipe tabacco (Manufactured by McClelland Tobacco Company, Kansas, Missouri, USA).
Floral	2 drops of Rose oil + 40 mL H2O / Aura Cacia pure essential Rose absolute fortifying in jojoba oil (Distributed by Frontier Natural Products CO-OP).
Taste / mouthfeel	
Sweet	15 g sucrose in 1000 ml water / C&H (Crockett, CA) Pure Cane Sugar granulated white
Salty	2 g salt in 1000 ml water / (Course Kosher Salt) Morton Salt (Chicago, IL)
Bitter	1.5 g caffeine in 1000 ml of water / (USP/FCC) Fisher Scientific (Fair Lawn, NJ)
Sour	3 g of tartaric acid in 1000 ml of water / (L-(+)-tartaric acid) Fisher Scientific (Fair Lawn, NJ)
Spicy	1 g of Cayenne pepper in 1000 ml of water / McCormick gourmet (Hunt Valley, MD)
Astringency	1 g of alum in 1000 ml of water (aluminium ammonium sulfate) McCormick (Hunt Valley, MD)
Viscous	4 g CMC in 1000 ml of water / (Carboxymethylcellulose sodium salt, medium viscosity, USP) Sigma-Aldrich (St. Louis, MO)
Hot	15% v/v EtOH / Seagram's extra smooth vodka (Lawrenceburg, IN)

Supplementary table 8. Attributes used in descriptive sensory analyses (DA) to rate the sensory profiles of Malbec wines from 2018 vintage and the reference standards used.

Attribute	**Reference standard**
Aromas	
Vanilla	3 drops of artificial vanilla in 40 mL of water / Vanilla Alicante 100 mL (Product of Argentina).
Banana	15 g banana in small pieces.
Spicy	0.5 g ground white pepper + 0.5 g ground black pepper + 4 grains of Jamaican pepper / ground white pepper and ground black pepper Alicante brand (Product of Argentina). Allspice obtained at the Central Market of Mendoza.
Jam	1 spoon of mixed jams / Marmalade Dulcor (Product of Argentina).
Chocolate	7 g of chocolate (2 squares) / Aguila, 42% cocoa (Product of Argentina).
Green pepper	5 g of green pepper in small pieces.
Dried plum	5 g of dried plum d'agen / Carrefour Argentina (Product of Argentina).

Earthy	5 g of peat for pots / Arhumus Black Fertile Fertile Soil.
Alcohol	15% v/v EtOH / Vodka Absolut (Sweden).
Grassy	5 g of fresh grass in small pieces
Herbaceous	5 g ground acacia leaf.
Leather	10 g of piece of leather tan
Red fruits	6 g of frozen strawberries + 6 g of frozen raspberries / Whole frozen strawberries and whole frozen raspberries "Green Life" (Product of Argentina).
Roses	3 red rose petals
Raisins	5 g of raisins cut in half / Sultanina grape (Jumbo Argentina)
Smoky	120 uL of liquid smoke in 50 mL of water / San Giorgio liquid smoke (Product of Argentina).
Humidity	moldy rag
Acetic	5 mL of Aceto / Aceto Dos Anclas (Product of Argentina).
Butter	10 gr of butter / Manteca La Serenisima (Product of Argentina).
Oak	Oak extract (20 gr oak chips in 250 mL for 1 hour) / French oak MT. ēvOAK (Product of France).
Taste / mouthfeel	
Sweet	20 g sucrose in 1000 ml of water / Pure white granulated ledesma cane sugar (Product of Argentina).
Bitter	1.5 g of caffeine in 1000 ml of water / Merck (Darmstadt, Germany).
Sour	3 g of tartaric acid in 1000 ml of water / (L-(+)-tartaric acid) Derivados vínicos (Mendoza, Argentina).
Salty	4.5 g salt in 1000 ml water / Sal Dos Anclas (Product of Argentina).
Astringency	1 g of alum in 1000 ml of water (aluminium ammonium sulfate) / Anedra Research AG S.A (Product of Argentina)
Spicy	3 g of hot pepper in 1000 ml of water / Ají picante Dos Anclas (Product of Argentina).
Viscosity	4 g of CMC in 1000 ml of water / CMC (Carboxymethylcellulose sodium salt) powdered (AEB.
Hot	15 % v/v de EtOH / Vodka Absolut (Sweden)
