# Supplementary material for: Tracing the origin of Argentine Malbec wines by sensometrics
Source: NPJ Sci Food. 2024 Feb 21;8:14. doi: 10.1038/s41538-024-00252-3 (PMC10879186; doi:10.1038/s41538-024-00252-3)
Supplement: Supplementary file 2 — Reporting summary [file 41538_2024_252_MOESM2_ESM.pdf]

nature portfolio

Corresponding author(s): Ariel Fontana

Last updated by author(s): Nov 20, 2023

Reporting Summary

Nature Portfolio wishes to improve the reproducibility of the work that we publish. This form provides structure for consistency and transparency in reporting. For further information on Nature Portfolio policies, see our Editorial Policies and the Editorial Policy Checklist.

Statistics

For all statistical analyses, confirm that the following items are present in the figure legend, table legend, main text, or Methods section.

n/a	Confirmed	
☐	☒	The exact sample size (*n*) for each experimental group/condition, given as a discrete number and unit of measurement
☐	☒	A statement on whether measurements were taken from distinct samples or whether the same sample was measured repeatedly
☐	☒	The statistical test(s) used AND whether they are one- or two-sided<br>*Only common tests should be described solely by name; describe more complex techniques in the Methods section.*
☐	☒	A description of all covariates tested
☐	☒	A description of any assumptions or corrections, such as tests of normality and adjustment for multiple comparisons
☐	☒	A full description of the statistical parameters including central tendency (e.g. means) or other basic estimates (e.g. regression coefficient) AND variation (e.g. standard deviation) or associated estimates of uncertainty (e.g. confidence intervals)
☐	☒	For null hypothesis testing, the test statistic (e.g. *F*, *t*, *r*) with confidence intervals, effect sizes, degrees of freedom and *P* value noted<br>*Give P values as exact values whenever suitable.*
☒	☐	For Bayesian analysis, information on the choice of priors and Markov chain Monte Carlo settings
☐	☒	For hierarchical and complex designs, identification of the appropriate level for tests and full reporting of outcomes
☐	☒	Estimates of effect sizes (e.g. Cohen's *d*, Pearson's *r*), indicating how they were calculated

Our web collection on statistics for biologists contains articles on many of the points above.

Software and code

Policy information about availability of computer code

Data collection	The 2017 panel data was collected on FIZZ software (ver. 2.51G; Biosystèmes, Couternon, France) and SOLDESA software was used for the 2018 panel.
Data analysis	Data was analyzed using the software platform R 3.2.2.

For manuscripts utilizing custom algorithms or software that are central to the research but not yet described in published literature, software must be made available to editors and reviewers. We strongly encourage code deposition in a community repository (e.g. GitHub). See the Nature Portfolio guidelines for submitting code & software for further information.

Data

Policy information about availability of data

All manuscripts must include a data availability statement. This statement should provide the following information, where applicable:

- Accession codes, unique identifiers, or web links for publicly available datasets
- A description of any restrictions on data availability
- For clinical datasets or third party data, please ensure that the statement adheres to our policy

The datasets generated during and/or analysed during the current study are available from the corresponding author on reasonable request.

Research involving human participants, their data, or biological material

Policy information about studies with human participants or human data. See also policy information about sex, gender (identity/presentation), and sexual orientation and race, ethnicity and racism.

Reporting on sex and gender	The results obtained do not apply to any particular sex or gender. Study participants were invited based on their availability. No personal identifying information is disclosed in this study. Participation was entirely voluntary for the panelists. Panelists could participate without force, fraud, deception, duress, coercion, or undue influence and retained the right to withdraw from the study at any point without any negative consequences. Panelists who consented to participate were provided with a copy of the "Permission to Participate in a Human Research Study" information sheet.
Reporting on race, ethnicity, or other socially relevant groupings	No information on race, ethnicity or other socially relevant groupings is reported. All participants were over 21 years of age, no participant was pregnant or breastfeeding, and no participant had medical, personal, or religious reasons that prevented them from consuming flavored beverages or wine. None of the following populations were included: - Adults who cannot give consent. - People who are not yet adults (babies, children, adolescents) - Pregnant women - Prisoners
Population characteristics	See above
Recruitment	For the 2017 panel held at UC Davis in Davis, California, USA, potential panelists were contacted using databases containing individuals who had previously granted permission to be approached, as well as department-maintained listservs. (Our own database, referenced in IRB protocol number 883721-1, was utilized.) Additionally, colleagues had the discretion to forward our recruitment email to their respective databases. Regarding the 2018 panel conducted at the INTA Sensory Lab in Mendoza, Argentina, participants were recruited using the lab's database and via social media platforms. Participants interested in sensory tasks and methodologies related to wine were required to be over 21 years old and not pregnant. Their age was verified with a photo ID before they participated in the panel. The purpose of the study was clearly explained to the participants, including the nature and objectives of the research, as well as the procedures that would be followed. They were informed about any common or significant discomforts and risks, along with the anticipated benefits.
Ethics oversight	The sensory panel 2017 conducted at UCDavis was reviewed and approved by an Institutional Review Board ("IRB"). The 2018 sensory panel, carried out at INTA, was approved by members of the INTA sensory analysis laboratory, Mendoza, Argentina experimental station.

Note that full information on the approval of the study protocol must also be provided in the manuscript.

Field-specific reporting

Please select the one below that is the best fit for your research. If you are not sure, read the appropriate sections before making your selection.

☐ Life sciences ☒ Behavioural & social sciences ☐ Ecological, evolutionary & environmental sciences

For a reference copy of the document with all sections, see nature.com/documents/nr-reporting-summary-flat.pdf

Behavioural & social sciences study design

All studies must disclose on these points even when the disclosure is negative.

Study description	The study is classified as quantitative and employs descriptive sensory analysis to evaluate wines from different regions of Mendoza across three vintages. In this approach, a panel of trained judges rate wines on sensory characteristics such as taste, aroma, and mouthfeel. Panelist rate each sensory attribute on unstructured line scales anchored on the left side of the scale with 'not present' and on the right side of the scale 'very intense'. The line scale ratings translate into numerical data, and all samples are replicated, allowing statistical analysis to determine significant differences between products and define a product's sensory profile.
Research sample	Panelists were recruited through advertising within the University. For the 2017 vintage wines, a total of 10 panelists (7 women and 3 men) participated, ranging in age from 21 to 55 years. For the 2018 vintage wines, 14 panelists (9 women and 5 men) were recruited, aged 24 to 59 years, many with prior experience in wine descriptive analysis. Study participants were invited based on their availability and interest. No personal identifying information is disclosed in this study.
Sampling strategy	The number of participants involved in the study was determined according to previous literature. In the case of descriptive sensory analysis, more than 8-15 participants are recommended. The procedure involved finding consent sensory attributes of the wines and training the participants on various sensory descriptors using aroma, taste, and mouthfeel reference standards beforehand. The descriptive analysis aims to have a common vocabulary to describe the wines. Each panelist was then required to evaluate wine samples blind three times. The glasses were labeled with random 3-digit codes, and the samples' presentation order was randomized

	using a modified Williams Latin Square design.
Data collection	The 2017 panel data was collected on FIZZ software (ver. 2.51G; Biosystèmes, Couternon, France), and SOLDESA software was used for the 2018 panel. Data collected in the 2017 panel using the FIZZ software were used on a computer. In the case of the 2018 panel using SOLDESA software, they were using each participant's cell phone.
Timing	The 2017 panel, held at UC Davis in California, USA, involved participants from October 9, 2017, to November 30, 2017.<br>The 2018 panel, held at INTA in Mendoza, Argentina, involved participants from September 27, 2018, to December 3, 2018.
Data exclusions	No data were excluded from the analyses. We found no reasons based on our exclusion criteria that required elimination of any data set.
Non-participation	In the panel conducted at UC Davis in 2018, there were four declined participation. The reasons provided by these participants for not continuing in the study were related to time availability issues for participating in the panel. Regarding the panel carried out at INTA, Mendoza in 2017, there was one dropout, who also cited scheduling conflicts as the reason for their withdrawal from the study
Randomization	No experimental groups were allocated for this study

Reporting for specific materials, systems and methods

We require information from authors about some types of materials, experimental systems and methods used in many studies. Here, indicate whether each material, system or method listed is relevant to your study. If you are not sure if a list item applies to your research, read the appropriate section before selecting a response.

Materials & experimental systems

n/a	Involved in the study
☒	☐ Antibodies
☒	☐ Eukaryotic cell lines
☒	☐ Palaeontology and archaeology
☒	☐ Animals and other organisms
☒	☐ Clinical data
☒	☐ Dual use research of concern
☒	☐ Plants

Methods

n/a	Involved in the study
☒	☐ ChIP-seq
☒	☐ Flow cytometry
☒	☐ MRI-based neuroimaging

Plants

Seed stocks	*Report on the source of all seed stocks or other plant material used. If applicable, state the seed stock centre and catalogue number. If plant specimens were collected from the field, describe the collection location, date and sampling procedures.*
Novel plant genotypes	*Describe the methods by which all novel plant genotypes were produced. This includes those generated by transgenic approaches, gene editing, chemical/radiation-based mutagenesis and hybridization. For transgenic lines, describe the transformation method, the number of independent lines analyzed and the generation upon which experiments were performed. For gene-edited lines, describe the editor used, the endogenous sequence targeted for editing, the targeting guide RNA sequence (if applicable) and how the editor was applied.*
Authentication	*Describe any authentication procedures for each seed stock used or novel genotype generated. Describe any experiments used to assess the effect of a mutation and, where applicable, how potential secondary effects (e.g. second site T-DNA insertions, mosiacism, off-target gene editing) were examined.*
